# Gestational Protein Restriction Increases Cardiac Connexin 43 mRNA levels in male adult rat offspring

**DOI:** 10.5935/abc.20170081

**Published:** 2017-07

**Authors:** Kamila Fernanda Rossini, Camila Andrea de Oliveira, Hércules Jonas Rebelato, Marcelo Augusto Marreto Esquisatto, Rosana Catisti

**Affiliations:** Programa de Pós Graduação em Ciências Biomédicas do Centro Universitário Hermínio Ometto FHO - UNIARARAS, Araras, SP - Brazil

**Keywords:** Pregnancy, Fetal Development, Connexin 43, Metabolism

## Abstract

**Background:**

The dietary limitation during pregnancy influences the growth and development of the fetus and offspring and their health into adult life. The mechanisms underlying the adverse effects of gestational protein restriction (GPR) in the development of the offspring hearts are not well understood.

**Objectives:**

The aim of this study was to evaluate the effects of GPR on cardiac structure in male rat offspring at day 60 after birth (d60).

**Methods:**

Pregnant Wistar rats were fed a normal-protein (NP, 17% casein) or low-protein (LP, 6% casein) diet. Blood pressure (BP) values from 60-day-old male offspring were measured by an indirect tail-cuff method using an electro sphygmomanometer. Hearts (d60) were collected for assessment of connexin 43 (Cx43) mRNA expression and morphological and morphometric analysis.

**Results:**

LP offspring showed no difference in body weight, although they were born lighter than NP offspring. BP levels were significantly higher in the LP group. We observed a significant increase in the area occupied by collagen fibers, a decrease in the number of cardiomyocytes by 10^4^ *µ*m^2^, and an increase in cardiomyocyte area associated with an increased Cx43 expression.

**Conclusion:**

GPR changes myocardial levels of Cx43 mRNA in male young adult rats, suggesting that this mechanism aims to compensate the fibrotic process by the accumulation of collagen fibers in the heart interstitium.

## Introduction

Maternal dietary restriction is a recognized cause of mortality at birth^1^ and low-birth weight.^2^ The concept of "programming" is used to associate prenatal events to changes in fetal growth that may become pathological in adulthood.^3,4^ Although molecular and physiological changes resulting from nutritional imbalance during pregnancy allow the offspring to survive, the long-term cardiovascular effects imposed by these changes promote structural modifications and changes in components of the renal, respiratory, endocrine, and central nervous systems.^5,6^ Recent data have shown bioenergetic changes in liver mitochondria of 30-day-old pups born from mothers undergoing protein restriction during gestation.^7^ In addition, gestational protein restriction (GPR) has been shown to be an important risk factor for cardiovascular disorders later in life.^8^

During electrical activation of the heart, all myocytes are individually activated by currents flowing through intercellular junctions. In the cardiovascular system, these gap junctions include one or more of four connexins - namely, Cx37, Cx40, Cx43, and Cx45 - that work together during the initial cardiovascular development.^9^ Gap junctions also ensure the mechanical and electrical communication between different types of muscle cells.^10^ This role is crucial in the heart since proper ejection of blood to the circulation depends necessarily on a coordinated contraction of both atrial and ventricular cardiomyocytes.^11,12^ Pathological conditions such as diabetes and hypertension are associated with deletions and changes in the regulation of connexin expression^13^ while connexin genes may have deleterious effects on cardiac function.^14^

The aim of this study was to evaluate the effects of GPR on cardiac structure in male rat offspring at day 60 (d60) after birth. We specifically investigated their blood pressure (BP) values during the 8th week of life, morphological and morphometric parameters of left ventricular cardiomyocytes, and Cx43 mRNA levels. Our choice to study the molecular profile of Cx43 was based on the fact that this is the most abundant and expressed connexin in the heart. This is the first study describing the cardiac expression of this gene in rats subjected to GPR.

## Methods

### Animal care

All experiments were conducted in strict agreement with the Guide for the Care and Use of Laboratory Animals and approved by the local Animal Care and Use Committee (Permit Nº. 056/2014). Ten-week-old virgin female Wistar rats weighing 180 to 250 g were mated with males. After confirming the pregnancy with observation of sperm in a vaginal smear (day 1 of pregnancy), we randomly allocated the pregnant rats (n = 12) on individual cages to receive an isocaloric and normal sodium semisynthetic diet (AIN 93G, Pragsoluções, Jau, SP, Brazil) with a normal protein content (17% casein, normal-protein [NP] group, n = 6, numbered from 1 to 6: 1NP to 6NP) or a low protein content (6% casein, low-protein [LP] group, n = 6, numbered from 1 to 6: 1LP to 6LP) (Table 1), as previously described.^7,15^ The animals were maintained at a controlled temperature (21 ± 1ºC) on a 12-h light/dark cycle, with free access to water until they delivered pups at 22 days of gestation. The anogenital distance was measured in all pups^16^ and litters were culled to a maximum of 8 males pups to minimize variation in nutrition during the suckling period. All liveborn male offspring of each mother were used in the experiments. When the number of male pups was less than 8, the number was increased by female pups until this value was reached. After weaning, the pups were housed in cages for a maximum of 4 animals, numbered and identified according to their affiliation. The number of cages followed the identification number of the mothers (from 1 to 6, NP or LP). When the number of male pups of each mother exceeded four, the cages were identified by the number of the mother plus the letters A or B. The identification of the pups in the cages was done by the marking on the tail: 1st (without tail marking), 2nd (one tail trace), 3rd (two tail traces) and 4th (three tail traces). All the animals were identified by this method and the total number of male rats was 21 for the NP group and 37 for the LP group. They received *ad libitum* water and a standard laboratory diet (21.6% protein and 4.0% lipid, Nuvilab CR-1, Nuvital, Colombo, PR, Brazil). On the 8th week of life, their BP levels were measured and, after anesthesia with ketamine (75 mg·kg^−1^ body weight, i.p.) and xylazine (10 mg·kg^−1^ body weight, i.p.), their hearts were removed for analysis. The hearts were weighed, and fragments from the middle third of the left ventricles were processed for morphological and molecular analyses. Twelve animals (NP, n = 6; LP, n = 6; 1 male rat for each of the mothers, randomly) were perfused for measurement of the cross-sectional area of the cardiomyocytes.

**Table 1:** Composition of the diets fed to the pregnant rats: normal protein (NP, 17%) and low protein (LP, 6%)^7,15^

g/kg	NP (17%)	LP (6%)
Cornstarch	397	480
Casein (84%)	202	71.5
Dextrin (90-94%)	130.5	159
Sucrose	100	121
Soybean oil	70	70
Fiber	50	50
Salt mixture (AIN 93 GMX)	35	35
Vitamin mix (AIN 93 VX)	10	10
L-cystine	3	1
Choline bitartrate	2.5	2.5

### Blood pressure measurement

Systemic arterial pressure was measured in conscious 7- and 8-week-old rats (LP, n = 12; NP, n = 12; 2 rats for each of the mothers, randomly) by an indirect tail-cuff method using an electrosphygmomanometer combined with a pneumatic pulse transducer/amplifier (IITC Life Science Inc., CA, USA). This indirect approach allowed repeated measurements with a close correlation (correlation coefficient = 0.975) to direct intra-arterial recording. The mean of three consecutive readings represented the BP level of the animal.

### Tissue collection: histology and morphometric analysis

Hearts were removed and longitudinally sectioned in the middle region in two halves. For histological analysis, the upper and lower halves of the six hearts from each experimental group animal were fixed using Millonig's solution and treated for paraffin embedding. Six-micrometer-thick sections were obtained from each blocked tissue from the middle region and stained with toluidine blue (TB) and picrosirius-hematoxylin (PH). Three of the sections stained with TB were used for cardiomyocyte counting (number per 10^4^ *µ*m^2^) and three other sections stained with PH were used for quantification of collagen fibers using polarization microscopy (% of birefringence area in 10^4^ *µ*m^2^). Five representative fields obtained from each longitudinal section of the left ventricle of each rat (150,000 *µ*m^2^ of total area by animal heart) were analyzed by light microscopy (Leica DM 2000 Photomicroscope) and captured with a digital Leica DFC 425 digital camera (Leica Microsystems, Wetzlar, Germany). Each digital image was photographed with the × 40 objective and formatted at fixed pixel density (8 × 10 inches at 150 dpi) using Sigma Scan Pro (v.6.0). At each digital image, the cardiomyocytes were counted following recommendation by Olivetti et al.^17^ and the area of birefringent collagen fibers was calculated as described by Mendes et al.^18^ For the analyses, the investigators were blinded to the group allocation.

### Measurement of cardiomyocyte cross-sectional area

The animals were anesthetized and perfused by the left carotid artery with saline containing heparin (5%) for 15 min and subsequently with 0.1 M phosphate buffer (pH 7.4) containing 4% (w/v) paraformaldehyde for 25 min. After perfusion, myocardial tissue samples were obtained from the septum and free wall of the middle part of the left ventricle and fixed in 4% phosphate-buffered formalin during 24 h for paraffin embedding. Five-micrometer-thick sections were cut from the blocked tissue and stained with hematoxylin-eosin (HE). The cross-sectional area of the cardiomyocytes was determined in at least 100 myocytes per slide stained with HE. The measurements were performed under a Leica DM 2000 microscope (x40 magnification lens) attached to a digital camera (Leica DFC 425, Leica Microsystems, Wetzlar, Germany) and connected to a personal computer equipped with the image analyser software Image J (National Institutes of Health, Bethesda, MD, USA). The cardiomyocyte area was measured with a digitizing pad, and the selected cells were transversely cut with the nucleus clearly identified in the center of the myocyte.^19^

### RNA isolation and semiquantitative reverse transcriptase-polymerase chain reaction (RT-PCR)

Total RNA was isolated from ~100-mg samples of left ventricular tissue with the TRIzol® reagent (Invitrogen, CA, USA) and digested with DNAse I, Amplification Grade (Invitrogen) according to the manufacturer's instructions. RNA concentration was determined by measuring UV absorbance at 260 nm using a spectrophotometer, and integrity was confirmed by formaldehyde gel electrophoresis. Samples of total RNA were stored at -80ºC until further use for analysis. cDNA was synthesized from 2 *µ*g of RNA in the presence of dithiothreitol, dNTP, random primers, RNAseOUT, and SuperScript™ II Reverse Transcriptase (Invitrogen) in a final volume of 20 *µ*L. Semiquantitative analysis of Cx43 mRNA expression was performed by RT-PCR in a final volume of 25 *µ*L containing 1 *µ*L of cDNA, 1.6 mM of MgCl_2_, 200 *µ*M of each dNTP, 0.2 ρmol of each primer, and 0.04 U of Taq DNA polymerase (Invitrogen, Itapevi, SP, Brazil). Cx43 was amplified using gene-specific forward (5´-GATTGAAGAGCACGGCAAGG-3') and reverse (5´-GTGTAGACCGCGCTCAAG-3´) primers with an expected amplicon of 144 bp (Tm 58ºC). ACTB (β-actin) was used as a housekeeping gene (Tm 57ºC; forward primer 5´-AGAGGGAAATCGTGCGTGACA-3' and reverse primer 5´-CGATAGTGATGACCTGACCGTCA-3´) yielding an amplification product of 178 bp that was used to normalize the Cx43 mRNA levels.

The amplified products were separated on 1.5% agarose gel stained with ethidium bromide, visualized, and photographed with the gel documentation system Syngene G: Box®. The signal intensity of the bands was measured densitometrically using the Scion Image software. Each value was determined as the mean of three densitometric readings. The results are expressed as average ratios of the relative optical densities of Cx43 PCR products in relation to the β-actin gene.

### Data analysis

The results were analyzed using the GraphPad Prism software (GraphPad Software, Inc., La Jolla, CA, USA) and are reported as the mean ± standard deviation (SD) of the measurements from six different animals. In cases in which two groups were compared, we used unpaired Student's t-test with a significance level of 5% (p < 0.05). When appropriate, we used analysis of variance (ANOVA) followed by Tukey's post-hoc test.

## Results

### Characteristics of the animals

**Figure 1:**
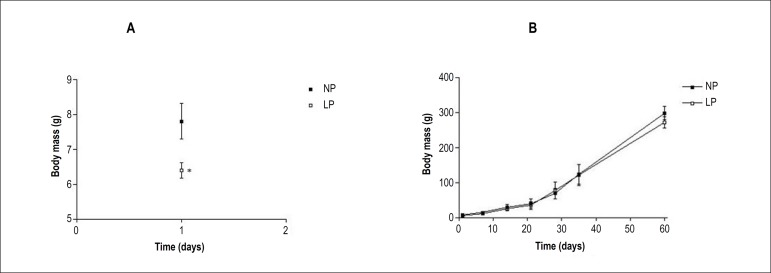
Effect of gestational protein restriction on the offspring weights. (A) weights on day 1 of male offspring of rats fed a normal-protein diet (NP, 17% protein, ■ symbol) or low-protein diet (LP, 6% protein, symbol □) during pregnancy (X ± SD, * p = 0.0048 versus NP); (B) growth curve of the offspring from the 1st to the 60th day after birth.

A previous analysis of the weight of pups on day 1 after birth (published by our research group and shown in Figure 1A^7,20^ for validation of the GPR model) showed that male offspring of mothers fed a low-protein diet (LP, n = 37, □ symbols, weight 6.40 ± 0.21 g) were significantly lighter than offspring of mothers fed a normal-protein diet (NP, n = 21, ■ symbols, weight 7.805 ± 0.51 g, * p = 0.0048, Figure 1A).^7,20^ Figure 1B shows the body weight gain of the animals over a period of 60 days after birth. At d60, there was no significant difference in weight between the NP and LP male offspring (LP, n = 37, □ symbols, final weight 271.8 ± 66.66 g; NP, n = 21, ■ symbols, final weight 298.3 ± 68.68 g).

### Effect of GPR on systemic arterial pressure and cardiac mass at d60

**Figure 2:**
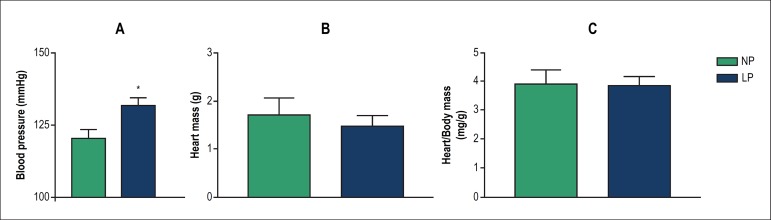
Effect of gestational protein restriction on the blood pressure levels and their cardiac mass of the offspring at d60. (A) blood pressure (mmHg); (B) cardiac mass (g); (C) ratio (mg/g) of the cardiac mass and body mass in young male offspring of rats fed a normal-protein diet (NP, full bar) or low-protein diet (LP, empty bars) (n = 12; X ± SD, * p = 0.021 versus NP).

The mean systemic BP values of the offspring at the 8th week of life are shown in Figure 2A. Values in the LP group (131.8 ± 2.7 mmHg) were significantly higher than those in the NP group (120.3 ± 3.33 mmHg, p = 0.021). Hearts isolated at d60 were quickly weighed after sacrifice, and their weights showed no significant difference (NP, 1.71 ± 0.34 g; LP, 1.48 ± 0.22 g), as shown in Figure 2B. Similarly, the ratio of heart tissue weight (mg) and body weight (g) (Figure 2C) showed no significant difference between groups (NP, 3.89 ± 0.48 mg/g; LP, 3.86 ± 0.28 mg/g).

### Effect of GPR on cardiac morphology

**Figure 3:**
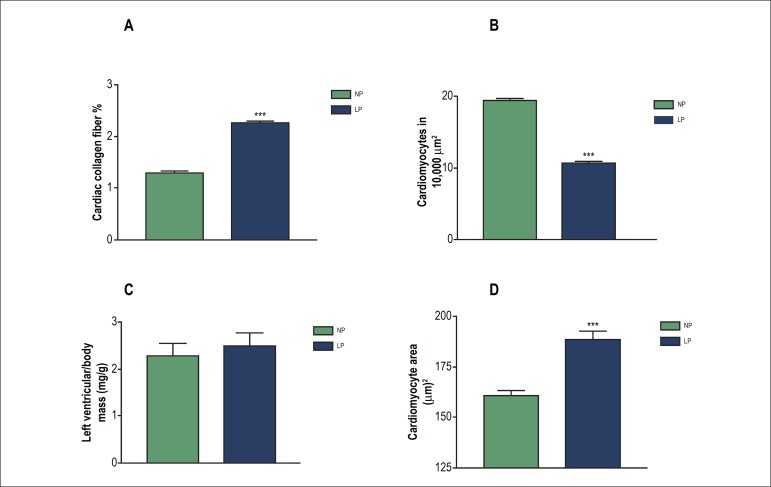
Effect of gestational protein restriction on cardiac morphometry at d60. (A) percentage of collagen fibers area/104 µm^2^ in left ventricular sections stained with picrosirius-hematoxylin (n = 6; *** p < 0.0001 versus NP); (B) number of myocytes/104 µm^2^ in left ventricular sections stained with toluidine blue (n = 6; * p < 0.0001 versus NP); (C) relationship between left ventricular weight and body mass (mg/g) at the age of 60 days in offspring of rats fed a normal-protein diet (NP, full bars) or low-protein diet (LP, empty bars); (D) cardiomyocyte cross-sectional area (µm^2^; X ± SD; n > 100 myocytes; *** p < 0.0001 versus NP).

Figure 3A shows the quantification of the area of collagen fibers in the heart of rats at d60. We observed a significant increase in the collagen fiber area in the heart of LP animals compared with NP ones. Morphometric analysis by TB staining allowed quantification of the number of myocytes present in the heart of NP and LP male offspring. The results showed a significant decrease in the number of myocytes in the hearts of LP offspring when compared with NP ones (Figure 3B). After perfusion of some animals (n = 6), the left ventricles were collected, weighed and processed for quantitation of the cardiomyocyte area. The ratio of left ventricle weight (mg) and body weight (g) (Figure 3C) showed no significant difference in the NP (2.28 ± 0.25 mg/g) and LP (2.49 ± 0.27 mg/g) groups. As seen in Figure 3D, the area of myocytes was significantly larger in the LP group (188.2 ± 4.14 *µ*m^2^) compared with the NP group (160.8 ± 2.57 *µ*m^2^).

### Modulation of Cx43 in the heart

**Figure 4:**
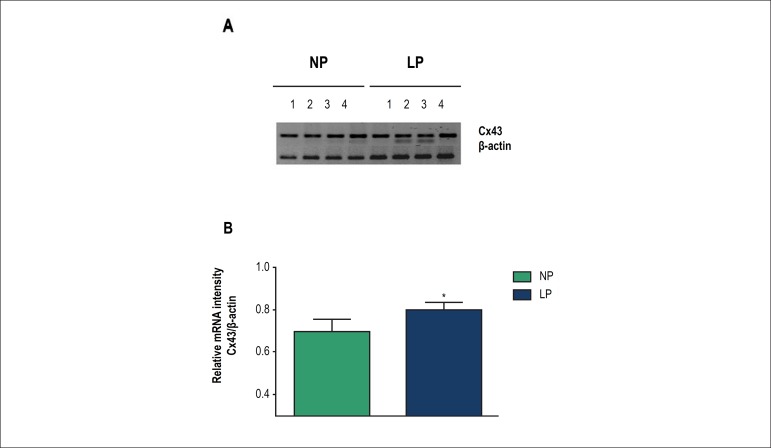
RT-PCR of Cx43 mRNA expression in the left ventricle (A) and densitometric analysis (B). Young male offspring of rats fed a normal-protein diet (NP, full bar) or low-protein diet (LP, empty bar) at d60 (n = 4; X ± SD of the optical density of Cx43 mRNA expression relative to β-actin; * p = 0.02 versus NP).

We collected left ventricular fragments for analysis of Cx43 expression. The values after densitometric analysis are shown in Figure 4. Compared with the NP group, the LP group showed significant increases in Cx43 mRNA levels (NP, 0.695 ± 0.058, n = 4, rats born to 4 different mothers; LP, 0.799 ± 0.032, n = 4; rats born to 4 different mothers).

## Discussion

As expected and described in the literature,^7,20,21^ offspring of rats that received a low-protein diet (LP group) were born lighter than offspring of rats fed a normal-protein diet (NP group). Fetal exposure to glucocorticoids (GC) has been proposed as one of the main risk factors for chronic diseases in adulthood.^22^ Exogenous or endogenous (maternal stress) fetal exposure to excess GC reduces fetal growth.^23-25^ During pregnancy, high levels of cortisol (in women)^26^ and corticosterone (in rats)^27^ are present in the maternal circulation.^24^ Several studies in rats have shown that maternal malnutrition in response to maternal stress increases corticosterone levels in the plasma, decreases placental expression of 11β-hydroxysteroid dehydrogenase type 2 (11β-HSD2), and increases placental expression of 11β-hydroxysteroid dehydrogenase type 1 (11β-HSD1).^24,28^ A study in sheep fetuses has shown that mineralocorticoid (MR) and GC (GR) receptors, as well as 11β-HSD1 are abundantly expressed in myocytes and cardiac blood vessels.^29^ The authors suggested that GC have access to both MR and GR in the fetal heart, and when GC plasma levels are elevated during a low-protein diet, the GC action in the cardiac MR and GR receptors also increases. GC could stimulate cardiac growth, either by hypertrophy or hyperplasia, or possibly even both. Cardiac hypertrophy could also result from high BP levels.^30^ At d60 in our study, the LP offspring had increased systolic BP levels in parallel to an increased area of cardiac collagen fibers. However, these changes were not sufficient to increase the heart weight, which would then characterize the changes as cardiac hypertrophy. Although the area of the cardiomyocytes increased, the number of cardiomyocytes reduced. This finding, observed in young male offspring hearts in our study, support the evidence of interstitial collagen deposition, a symptom of cardiac hypertrophy in response to hypertension in adult human hearts.^30^

The renin-angiotensin system (RAS) plays an important role in primary and secondary forms of hypertension. Components of the RAS, such as angiotensin-converting enzyme (ACE) and angiotensin II, are locally produced in cardiac tissues^31^ and are primary candidates for factors promoting remodeling, mainly cardiac myocyte hypertrophy and increased extracellular fibrosis, which lead to deterioration in cardiac function.^32^ Various experimental animal models have been developed to investigate the associations between fetal undernutrition and cardiovascular disease later in life,^33,34^ and a possible involvement of systemic RAS in the development of hypertension has been reported.^35,36^

The composition of the extracellular matrix in physiological and pathophysiological conditions can affect the degree of electrical coupling in cardiac myocytes.^37^ The conduction of electrical impulses in the heart is determined mainly by three key parameters: electrical coupling between cardiomyocytes, excitability of individual cardiomyocytes, and connective tissue architecture.^37^ These parameters of conduction are primarily mediated by Cx43, NaV1.5 sodium channels, and by the amount of collagen fibers, respectively. In cardiac arrhythmias,^38^ abnormalities in any of these driving parameters have frequently been observed. Cx43 is generally down-regulated, less phosphorylated, and/or redistributed at the intercalated discs along the lateral aspects of the cardiomyocyte.^14,39,40^ Our study provides the first evidence of increased Cx43 expression in rat hearts induced by GPR. Although our results are limited, we hypothesize that the increased deposition of collagen fibers in the heart associated with increased systolic BP lead to changes in the cardiac conduction of electrical impulses. In response to this injury and associated with the observed increased cardiomyocyte area, the preservation of cell-to-cell communication via upregulation of myocardial Cx43 may be attributed to a protective effect.

## Conclusion

Using a rat model of fetal protein restriction, we showed that GPR affects the organization and number of myocytes in the offspring heart and increases the amount of collagen fibers in the cardiac tissue, showing clearly a degenerative process compatible with fibrosis. This finding reinforces the association between maternal malnutrition with low birth weight and the risk of cardiovascular morbidity in adulthood. GPR increases the area of cardiomyocytes and expression of Cx43 in the myocardium of young adult male rats, suggesting that this mechanism aims to compensate the fibrotic process by the accumulation of collagen fibers in the heart interstitium.
